# Atypical Presentation of Peripheral Ossifying Fibroma in the Mandible

**DOI:** 10.7759/cureus.22375

**Published:** 2022-02-19

**Authors:** Vinod K Krishna, Senthilnathan Periasamy, Santhosh P Kumar, Swetha V Bhat

**Affiliations:** 1 Oral and Maxillofacial Surgery, Saveetha Dental College and Hospital, Chennai, IND

**Keywords:** inflammatory fibrous hyperplasia, recurrence, irritation fibromatosis, gingival hyperplasia, peripheral ossifying fibroma

## Abstract

Peripheral ossifying fibroma (POF) is a localized reactive enlargement of the gingiva often associated with the papilla and originate from underneath the periodontium. POF occurs predominantly in females, especially in the anterior maxillary region of young adults. The histopathological examination provides a confirmatory diagnosis of such lesions due to their heterogeneous clinical and radiographic characteristics. This case report describes an atypical presentation of POF in the anterior mandible in an adult male patient. Treatment consisted of complete surgical excision and gingival curettage resulting in uneventful healing during the postoperative follow-up period.

## Introduction

Gingiva in the oral cavity is constantly subjected to numerous stimuli resulting in numerous localized growths. Reactive gingival growth is the most common lesion in the oral cavity and usually exhibits an indolent behavior [1]. Ossifying fibromas are classified into two types: central and peripheral. The central ossifying fibroma arises from the endosteum or the periodontal ligament adjacent to the root apex causing expansion of the medullary cavity. Peripheral ossifying fibroma (POF) occurs exclusively on the soft tissues in the tooth-bearing areas of the jaws [2]. POF has a peak incidence in young and teenage individuals, with a predilection for females. Overall, 60% of POFs occur in the maxillary jaw, especially in the incisor-canine region. POF constitutes 9.6% of the gingival lesions, and it rarely occurs in the anterior mandibular region [3]. This case report describes an atypical presentation of POF in the anterior mandible in an adult male patient.

## Case presentation

A 41-year-old male patient reported to the Department of Oral and Maxillofacial surgery complaining of a swelling on the right side of his lower jaw for the past two years. History revealed that the swelling was initially the size of a pea and grew rapidly to the present size. The patient complained of difficulty in speech and mastication due to the swelling. The patient gave a history of a similar growth in the mandibular anterior region two years ago, which was excised and diagnosed as ossifying fibroma epulis. Medical history revealed that the patient was hypertensive and was consuming medications.

**Figure 1:**
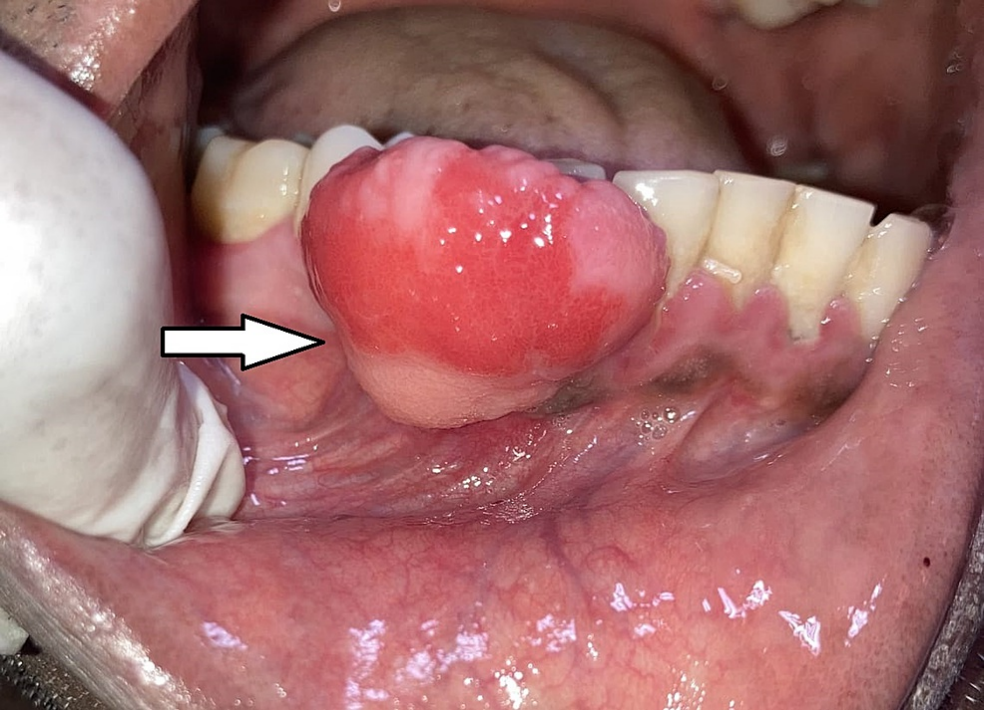
Intraoral view showing the lesion in the right anterior region of the mandible.

Extraoral examination revealed no gross asymmetry, but the patient’s lips were incompetent due to the presence of swelling. Regional lymph nodes were not palpable. Intraoral examination showed a pink, sessile, fibrous, exophytic mass extending from 42 to 45 region obliterating the buccal sulcus (Figure 1). The growth was non-tender and soft to firm in consistency, measuring 2.3 × 2.0 cm in size and protruding from the labial gingiva.

**Figure 2:**
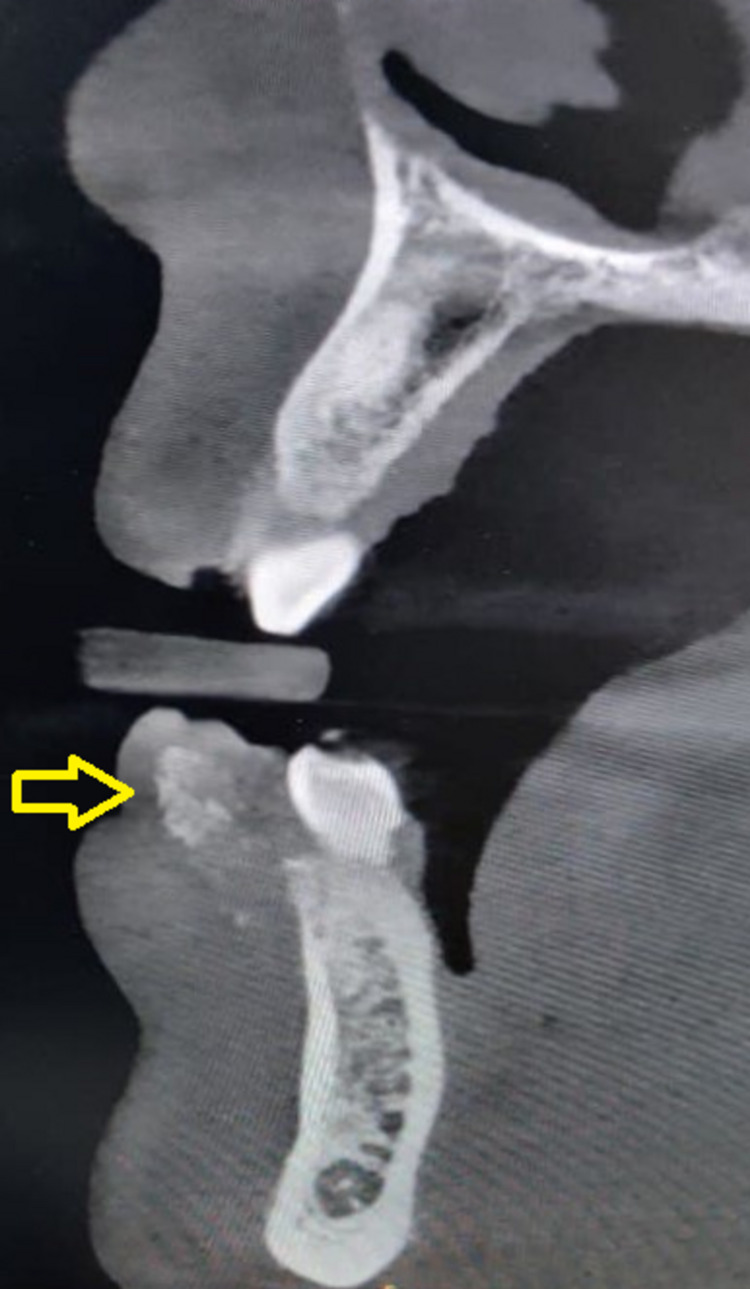
Cone-beam computed tomography showing soft tissue opacification in the right anterior mandible region.

Cone-beam computed tomography revealed opacification of the soft tissues in the right anterior mandible region, with no displacement, resorption, displacement of the tooth, or any bone loss in the associated region (Figure 2). Based on the history, clinical presentation, and radiological investigation, the lesion was provisionally diagnosed as POF and planned for excision.

**Figure 3:**
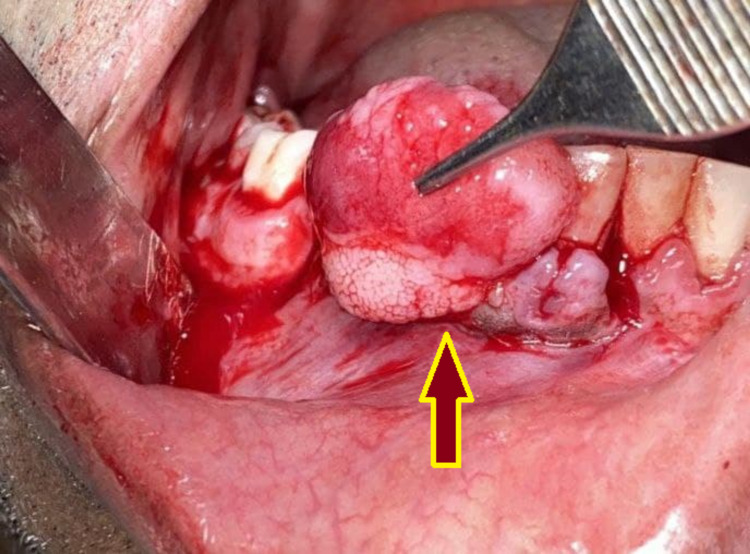
Displacement of the lesion from its attachments.

**Figure 4:**
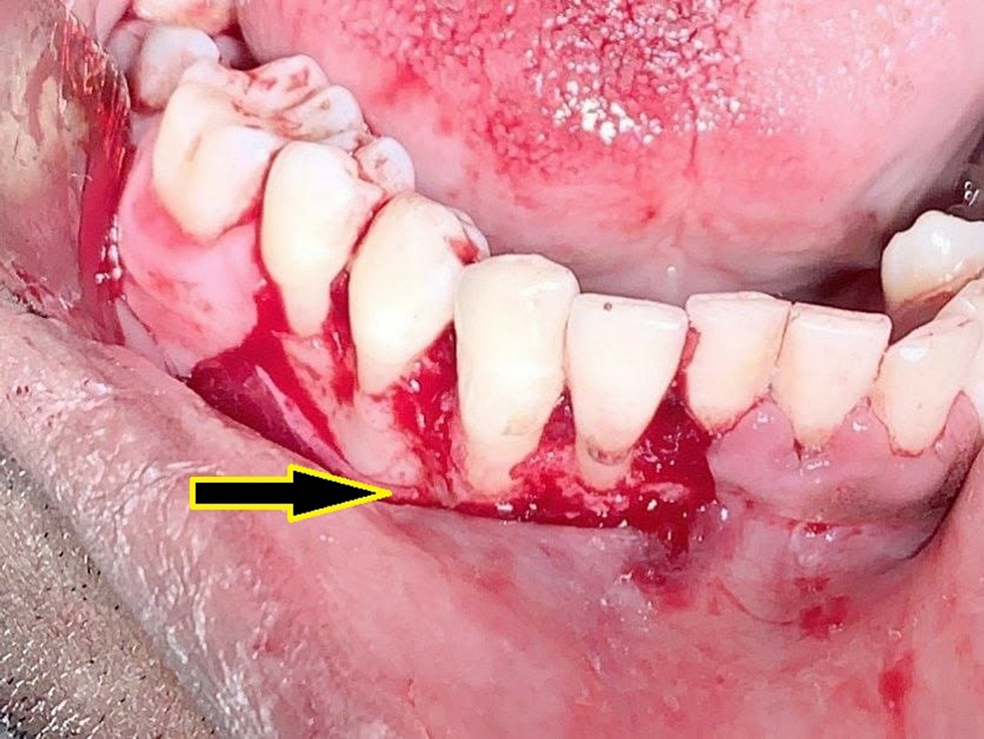
Surgical site after removal of the lesion and debridement.

Under local anesthesia, a crevicular incision was done for the involved teeth and the flap was elevated along with the lesion. The whole growth was excised through a wedge incision around the lesion (Figure 3), and thorough curettage was done in the depth of the lesion and surrounding tissues (Figure 4). After achieving hemostasis, a periodontal dressing was applied, and the patient was prescribed antibiotics, analgesics, and chlorhexidine mouthwash.

**Figure 5:**
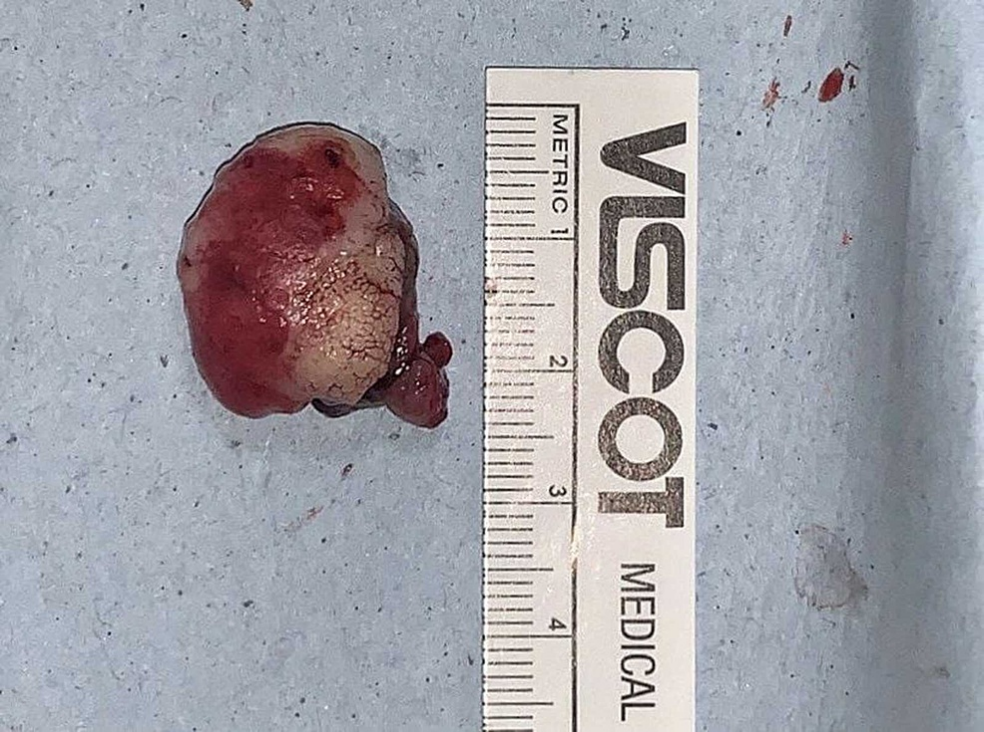
Excised specimen from the gingiva.

**Figure 6:**
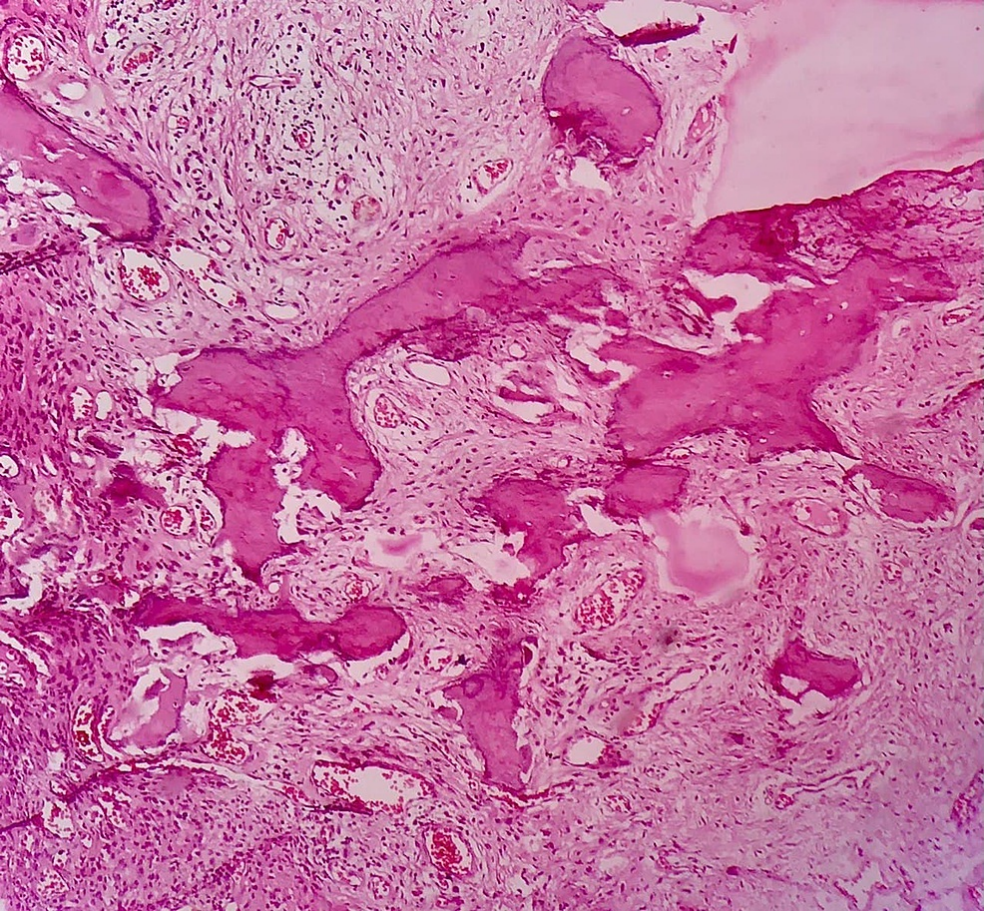
Microscopic examination revealing features suggestive of peripheral ossifying fibroma.

**Figure 7:**
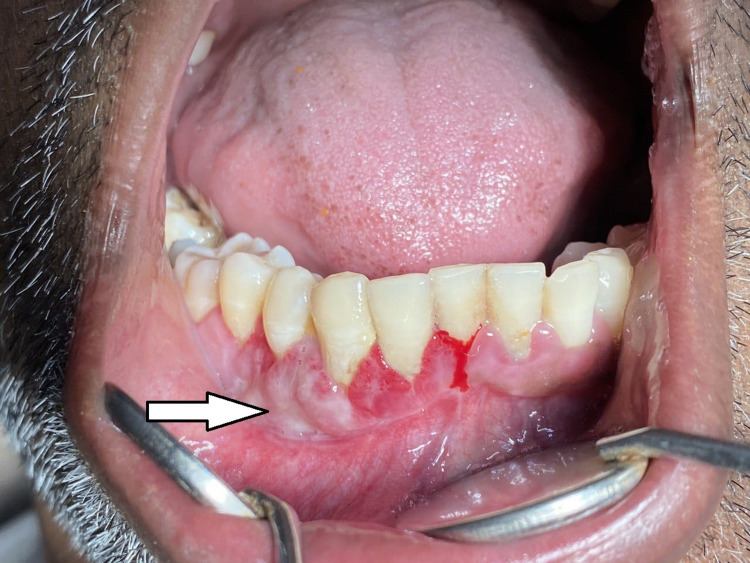
Uneventful wound healing in the immediate postoperative period.

The excised specimen was sent for histopathological examination (Figure 5). Histology revealed para-keratinized stratified squamous epithelium exhibiting pseudoepitheliomatous hyperplasia with few epithelial cell rests, presence of few eosinophilic mineralized areas of variable sizes showing trabeculae and osteocytes resembling bone with moderate chronic inflammatory cell infiltrate, engorged blood vessels, and endothelial cell proliferation. The section also showed mature connective tissue stroma with areas of fibrosis and the presence of myxoid appearance confirming the diagnosis of POF (Figure 6). Wound healing was uneventful in the immediate postoperative period (Figure 7). On clinical examination during the six-month postoperative follow-up period, wound healing was good without any recurrences.

## Discussion

The connective tissue of the periodontium of the oral cavity can exhibit distinctive focal overgrowths, corresponding to 90% of gingival biopsies [4]. Commonly occurring oral cavity lesions are inflammatory fibrous hyperplasia, pyogenic granuloma, POF, and giant-cell granuloma. POFs are frequent gingival overgrowths and are also known as ossifying fibroid epulis [5]. They are reactive in nature which occurs due to hyperplastic reaction to inflammation but are not neoplastic. Factors causing initiation and growth of POF are calculus, ill-fitting dentures, masticatory forces, food impaction, plaque, sharp restorations, oral microorganisms, trauma, and oral debris [6]. POF arises from the periodontal ligament as it exclusively develops in the gingival portion, as well as because of the presence of oxytalan fibers inside the mineralization of some lesions. It can also arise due to the maturation of pre-existing pyogenic granuloma or a peripheral giant cell granuloma. POF has a high potential for the formation of bone and cementum-like materials [7].

POF has a peak incidence in young and teenage individuals, with a female predilection. Overall, 60% of POFs occur in the maxillary jaw, especially in the incisor-canine region. Our case had an atypical presentation in the anterior mandible in the middle-aged male person. POF manifests clinically as well-demarcated gingival overgrowth, pink to red in color with an ulcerated surface, and the base of the lesions may be sessile or pedunculated. It usually does not blanch upon palpation and frequently originates from an interdental papilla with a size less than 2 cm in its greatest dimension [8]. Differential diagnosis includes pyogenic granuloma, peripheral odontogenic fibroma, chondrosarcoma or osteosarcoma, peripheral giant-cell granuloma, fibroma, and hemangioma.

Radiographic evaluation of these lesions exhibits radiopaque calcifications in soft tissues and sometimes with the presence of associated bone destruction. In only 5% of the cases, the radiographic appearance of tooth migration is seen [9]. In our patient, cone-beam computed tomography revealed a soft tissue opacification in the right anterior mandible, with no displacement, resorption, displacement of the tooth, or any bone loss in the associated region. Histologically, it appears as a non-capsulated mass consisting of stratified squamous epithelium and cellular fibroblastic connective tissue. Sometimes it exhibits the arbitrary distribution of calcifications in the connective tissue, which may lead to misdiagnosis [10].

Treatment of POF includes complete excision of the lesion with the underlying periosteum and periodontal ligament, as well as the removal of local irritants by thorough curettage to prevent relapse. Extraction of the teeth adjacent to the lesion is rarely required [11]. In untreated cases, the lesion may grow to an enormous size causing destruction of the surrounding bone [12]. Although most of the POF arises from the periodontal ligament, there are reports of these lesions occurring in edentulous jaws [13]. Incomplete excision of the lesion and the irritating factors are the common causes for recurrence of POF, which ranges from 8-20% [14]. In our patient, a similar lesion was excised two years before, thus representing a recurrent lesion. Due to the high relapse rate of these lesions, periodic review and long-term postoperative follow-up are required.

## Conclusions

Diagnosing POF is challenging as it exhibits features similar to other conditions in the oral cavity. Careful clinical and radiographical examination correlated with histopathological findings is required for prompt diagnosis. POF should also be considered in the differential diagnosis of acute and big-sized lesions of the oral cavity. POFs are treated by complete surgical excision and gingival curettage, followed by good oral hygiene maintenance. Patients should be followed up for a longer period of time due to high relapse rates.
